# Liver microsomal lipid enhances the activity and redox coupling of colocalized cytochrome P450 reductase‐cytochrome P450 3A4 in nanodiscs

**DOI:** 10.1111/febs.14129

**Published:** 2017-06-30

**Authors:** Kang‐Cheng Liu, John M. X. Hughes, Sam Hay, Nigel S. Scrutton

**Affiliations:** ^1^ Centre for Synthetic Biology of Fine and Speciality Chemicals (SYNBIOCHEM) School of Chemistry Manchester Institute of Biotechnology The University of Manchester UK

**Keywords:** cytochrome P450, lipid, liver, nanodiscs, reductase

## Abstract

The haem‐containing mono‐oxygenase cytochrome P450 3A4 (CYP3A4) and its redox partner NADPH‐dependent cytochrome P450 oxidoreductase (CPR) are among the most important enzymes in human liver for metabolizing drugs and xenobiotic compounds. They are membrane‐bound in the endoplasmic reticulum (ER). How ER colocalization and the complex ER phospholipid composition influence enzyme activity are not well understood. CPR and CYP3A4 were incorporated into phospholipid bilayer nanodiscs, both singly, and together in a 1 : 1 ratio, to investigate the significance of membrane insertion and the influence of varying membrane composition on steady‐state reaction kinetics. Reaction kinetics were analysed using a fluorimetric assay with 7‐benzyloxyquinoline as substrate for CYP3A4. Full activity of the mono‐oxygenase system, with electron transfer from NADPH via CPR, could only be reconstituted when CPR and CYP3A4 were colocalized within the same nanodiscs. No activity was observed when CPR and CYP3A4 were each incorporated separately into nanodiscs then mixed together, or when soluble forms of CPR were mixed with preassembled CYP3A4‐nanodiscs. Membrane integration and colocalization are therefore essential for electron transfer. Liver microsomal lipid had an enhancing effect compared with phosphatidylcholine on the activity of CPR alone in nanodiscs, and a greater enhancing effect on the activity of CPR‐CYP3A4 nanodisc complexes, which was not matched by a phospholipid mixture designed to mimic the ER composition. Furthermore, liver lipid enhanced redox coupling within the system. Thus, natural ER lipids possess properties or include components important for enhanced catalysis by CPR‐CYP3A4 nanodisc complexes. Our findings demonstrate the importance of using natural lipid preparations for the detailed analysis of membrane protein activity.

**Abbreviations**
2‐OH‐Mito‐E^+^: 2‐hydroxyethidium cation of Mito‐HE
7‐BQ: 7‐benzyloxyquinoline
7‐HQ: 7‐hydroxyquinoline
CHAPS: 3‐[(3‐cholamidopropyl)dimethylammonio]‐1‐propanesulfonate
CPR: cytochrome P450 reductase
CYP: cytochrome P450
DTT: dithiothreitol
EDTA: ethylenediaminetetraacetic acid
ER: endoplasmic reticulum
FMN: riboflavin‐5′‐phosphate
His‐tag: poly‐histidine affinity tag
IPTG: isopropyl β‐d‐1‐thiogalactopyranoside
*k*_cat_: catalytic constant
*K*_M_: Michaelis constant
LB: ‘Lauria–Bertani’ broth
MAD: membrane anchor domain
Mito‐HE: hydroethidine linked by a hexyl carbon chain to a triphenylphosphonium group (‘MitoSOX Red’)
MSP: membrane scaffold protein
MTT: thiazolyl blue tetrazolium bromide
NADPH: β‐nicotinamide adenine dinucleotide phosphate
Ni‐NTA: nickel nitrilotriacetic acid
OmpA: *Escherichia coli* outer membrane protein A
PA: phosphatidic acid
PC: phosphatidylcholine
PE: phosphatidylethanolamine
PI: phosphatidylinositol
POPC: 2‐oleoyl‐1‐palmitoyl‐sn‐glycero‐3‐phosphocholine
PS: phosphatidylserine
SM: sphingomyelin
TB: Terrific Broth
Tris: tris(hydroxymethyl)aminomethane
ΔN‐CPR: 60 amino acid N‐terminally truncated CPR

## Introduction

Cytochrome P450 3A4 (CYP3A4) is the most abundant human cytochrome P450 (CYP). Along with its redox partner, NADPH‐dependent cytochrome P450 reductase (CPR), it is membrane‐bound in the endoplasmic reticulum (ER) and found predominantly in cells of the liver and small intestine. CYP3A4 has great pharmacological significance since it is responsible for the metabolism of about half of all medically prescribed drugs and many xenobiotic compounds 1, 2, 3. CYPs are haem protein mono‐oxygenases whose catalytic activity depends on the integrity of a multicomponent electron transfer chain. Eukaryotic CYPs in the ER depend for activity on electron transfer from NADPH via flavin adenine dinucleotide and riboflavin‐5′‐phosphate (FMN) moieties of the diflavin protein CPR, and the coordinated activity of these two proteins likely depends on their close association within the ER membrane. Although the fundamentally conserved reaction mechanism of this coupled enzyme system is understood in some detail 4, 5, 6, 7, the influence of the complex composition of the surrounding membrane on catalytic activity is poorly understood. Furthermore, comparative studies of CYP catalytic activity are complicated by the various effects of homotropic and heterotropic cooperativity of substrate binding, homomeric and heteromeric complex formation, and different CPR : CYP ratios 8, 9, 10.

Eukaryotic CPR and CYPs each have an N‐terminal membrane anchor domain (MAD) containing hydrophobic amino acids by which they are tethered to the ER 11, 12, 13. An intact MAD on CPR, as well as the association of phospholipid, have long been recognized as requirements for the coupled activity of the two enzymes in reconstituted mammalian systems. Early methods to isolate mammalian CPR involved treatment of microsomes with trypsin, yielding a proteolytically cleaved form of the enzyme which lacked the MAD. This form of CPR was able to reduce cytochrome *c*, but failed to interact functionally with its natural CYP redox partners (reviewed in ref. 14). Only when CPR was purified intact from microsomes using detergent was it first possible to reconstitute a functional CYP‐mediated mono‐oxygenase system. The activity also required the inclusion of phospholipid, the essential component of which was shown to be phosphatidylcholine (PC) 15, 16. An intact MAD to tether the CPR to the phospholipid membrane therefore appeared to be essential for reconstitution of the activity. However, the MAD is not merely required to tether CPR to the membrane, since competitive peptide binding indicated that reconstitution of activity required the direct interaction of the MAD of CPR with CYP 11.

Although the MAD could be the sole determinant of CPR binding to the ER, this is almost certainly not the case for CYP. Molecular dynamics simulations and other studies suggest that CYP makes extensive contact to the membrane with the face distal to the side of CPR interaction, and that it could sit partly immersed within the membrane. This intimate embedding within the membrane is possibly essential to the integrity of access tunnels through the protein structure by which substrates gain entry to the active site 13, 17, 18, 19. Many CYP substrates are lipophilic, thus their access to the CYP active site is likely to be through the membrane 20, 21. This embedding within the membrane may explain why integrity of the MAD of CYP is not necessarily essential for reconstitution of the coupled mono‐oxygenase system 22, 23, 24.

The ER has a complex phospholipid composition and has structural heterogeneity, and studies of CPR and CYP1A2 from the ER of rabbit liver show that these enzymes copurify with lipid microdomain fractions enriched in certain phospholipids 25, 26. How and to what extent different phospholipids influence the activity of the mono‐oxygenase systems is not clear. The influence of different phospholipids has been studied by reconstitution in phospholipid vesicles. Generally it has been found that increasing the proportion of anionic phospholipid in lipid mixtures stimulates activity, and that specific anionic phospholipids, such as phosphatidic acid (PA), can have greater stimulatory effect. Enhanced activity has been proposed to be due to better binding and deeper insertion of protein within the membrane, and closer interaction between CPR and CYP promoting more effective coupling for electron transfer 27, 28, 29, 30, 31. Furthermore, the stimulatory effect of specific anionic phospholipids has been shown to be dependent on an intact MAD on the CYP 23, 24.

When CYP3A4 was incorporated with CPR into vesicle membranes of varied composition, maximal product formation rate was observed in vesicles composed of liver microsomal phospholipid. Compared with PC alone, a PC/PS (phosphatidyl serine) mixture of 3 : 1 enhanced the rate by about twofold, whereas liver microsomal phospholipid enhanced the rate by about fivefold. Moreover, this maximal rate was accompanied by a high degree of electron transfer coupling 32. Not all CYPs, however, show the same responses. Enhancement of CYP3A1 reconstituted activity, which is highly dependent on coincorporation of cytochrome *b*5 in membrane vesicles, varied with different phospholipids according to the inclusion of detergents in the reaction. Detergent possibly ameliorated the effects of protein aggregation. Activity in liver microsomal phospholipid was enhanced by detergent, but to no greater extent than with PC. Furthermore, the importance of unsaturated fatty acid in PC was shown 33.

Phospholipid bilayer nanodiscs are disc‐shaped membrane bilayers bound round the circumference by a double protein belt derived from apolipoprotein A1. As a means to study membrane protein incorporated in a native‐like membrane in an aqueous environment, they offer the advantage over lipid vesicles of being more stable, and of being monodisperse and homogeneous in size, therefore affording greater control of stoichiometry and of avoiding the possibly complicating effects of aggregation 34, 35, 36. The properties of human CPR and CYP3A4 have been studied in nanodiscs, both singly 37, 38, 39, 40, 41 and together as a functional pair 42, 43. However, nanodiscs have been little used to study the influence of different phospholipid composition on the activity of the enzymes. When CPR was incorporated monomerically in nanodiscs, changing the phospholipid composition from pure PC to a 50% PS/PC mixture caused flavin redox potential changes that were suggested could favour electron transfer from NADPH to CYP3A4. The redox potential difference across the flavin couple was increased in the PS/PC mixture compared with PC alone, providing greater impetus (driving force) for electron transfer. Thus, an increase in the content of anionic phospholipid was proposed to have an enhancing effect 37. Membranes of mixed PS/PC (30% PS), furthermore, enhanced the activity of the CPR‐CYP3A4 complex in nanodiscs, improving both the coupling and the NADPH‐dependent substrate conversion rates (for two of the three substrates tested). It was suggested that this enhancement could also be due to effects on redox potential 43. A drawback of these studies, however, was that none of these phospholipid mixtures resembled the natural composition of the ER.

No studies of CPR/CYP function so far have been made using nanodiscs of natural ER membrane composition. In our investigation we have chosen to exploit nanodiscs to study the influence of physiologically relevant membrane composition on the activities of CPR and CYP3A4. Through better control of the stoichiometry of membrane protein insertion into the nanodiscs, we hoped to avoid some of the inconsistency and variability due to aggregation and multimeric complex formation possibly inherent in previous studies involving lipid vesicles. For the assay of CYP3A4 mono‐oxygenase activity, we have confined our investigation to a single substrate, 7‐benzyloxyquinoline (7‐BQ), the conversion of which to 7‐hydroxyquinoline (7‐HQ) can be measured conveniently by fluorimetry 44. We have studied the effects of different membrane compositions on the activity of CPR, both alone, and in combination with CYP3A4 as a functional monodisperse 1 : 1 complex. In order to be physiologically relevant, we have compared enzyme activities within membranes composed of lipid extracted directly from liver microsomes, with PC alone, and in the case of the coupled enzyme complex with a mixture of component phospholipids designed to mimic the known composition of the ER. We have determined steady‐state kinetic parameters in the various combinations, and investigated coupling in the dual enzyme system. We find that, compared with PC alone, liver microsomal lipid enhances the activities of both CPR and of the coupled enzyme pair, and furthermore liver microsomal lipid increases the extent of redox coupling during electron transfer between partner proteins.

## Results

### Assembly of phospholipid bilayer nanodiscs containing CPR and CYP3A4

Phospholipid bilayer nanodiscs were assembled essentially as previously described 45, incorporating CPR or CYP3A4 either singly, or together as described by Denisov *et al*. 42. Each nanodisc consists of two protein molecules derived from apolipoprotein A1, referred to as ‘membrane scaffold protein’ (MSP), arranged antiparallel to each other in the form of a double belt, within which the phospholipid bilayer membrane forms like the skin of a drum. Nanodiscs are assembled by mixing the constituent components together in appropriate ratio in detergent, then removing the detergent by adsorption to polystyrene beads. Combinations of gel filtration, affinity purification and nondenaturing and denaturing gel electrophoresis were used to demonstrate assembly and to purify nanodiscs containing inserted membrane protein. Premixing the assembly components in the appropriate ratio is important. Too great an excess of phospholipid can lead to the formation of lipid vesicles that can sequester membrane protein, and too little lipid can lead to protein aggregation.

**Figure 1:**
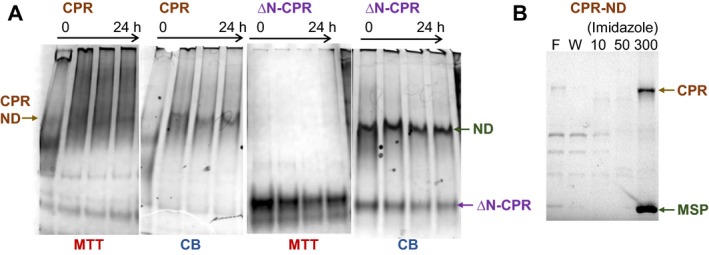
Assembly and purification of nanodiscs containing active CPR. (A) Nanodisc assembly mixtures composed with either full‐length (CPR) or N‐terminally truncated CPR (ΔN‐CPR), sampled over time from the start of detergent removal, resolved by native PAGE. Gels were stained with either MTT for CPR activity, or Coomassie Blue (CB) for total protein. CPR‐containing nanodiscs (CPR‐ND) and unloaded nanodiscs (ND) are indicated. (B) Enrichment of CPR‐nanodiscs by Ni‐affinity chromatography, samples resolved by SDS/PAGE. Flow‐through (F), wash (W), mm imidazole concentration in wash and eluate, CPR and membrane scaffold protein MSP1D1 (MSP) indicated.

The assembly of enzymatically active CPR in nanodiscs was demonstrated by native (nondenaturing) gel electrophoresis. Complexes within the gel which contained active CPR were identified by developing a staining procedure using the dye thiazolyl blue tetrazolium bromide (also known as methylthiazolyldiphenyl‐tetrazolium bromide or MTT). MTT is readily soluble and forms a pale yellow solution. NAD(P)H‐oxidoreductases catalyse the reduction of MTT to purple insoluble formazan 46. When gels were soaked in MTT solution to which NADPH was added, formazan formed in the bands that contained CPR. Nanodisc complexes containing CPR gave rise to formazan staining, confirming that active CPR was present in these complexes (visible half way down the gel, Fig. 1A, first panel). However, when an N‐terminally truncated form of CPR lacking the MAD was used in the assembly mixture instead of full‐length CPR, formazan staining was not associated with the nanodisc complexes but was associated with a faster migrating band corresponding to free CPR (visible near the bottom of the gel, Fig. 1A, third panel); nanodiscs were still able to form in the latter mixture (shown by Coomassie Blue staining of the MSP half way down the gel, Fig. 1A, fourth panel) but they were devoid of any active CPR. These results therefore show not only that active CPR could be assembled within the nanodiscs but also that the MAD of CPR is essential for assembly of CPR in the nanodiscs.

Nanodiscs were purified from the assembly mixture by adsorption to nickel‐resin of the N‐terminal poly‐histidine tag (His‐tag) contained on the MSP. CPR remained associated with the nanodiscs adsorbed to the resin, and was eluted together with the nanodiscs in 0.3 m imidazole (Fig. 1B). Further purification of the CPR‐nanodiscs from nanodiscs lacking CPR was not necessary for the kinetic studies described below.

**Figure 2:**
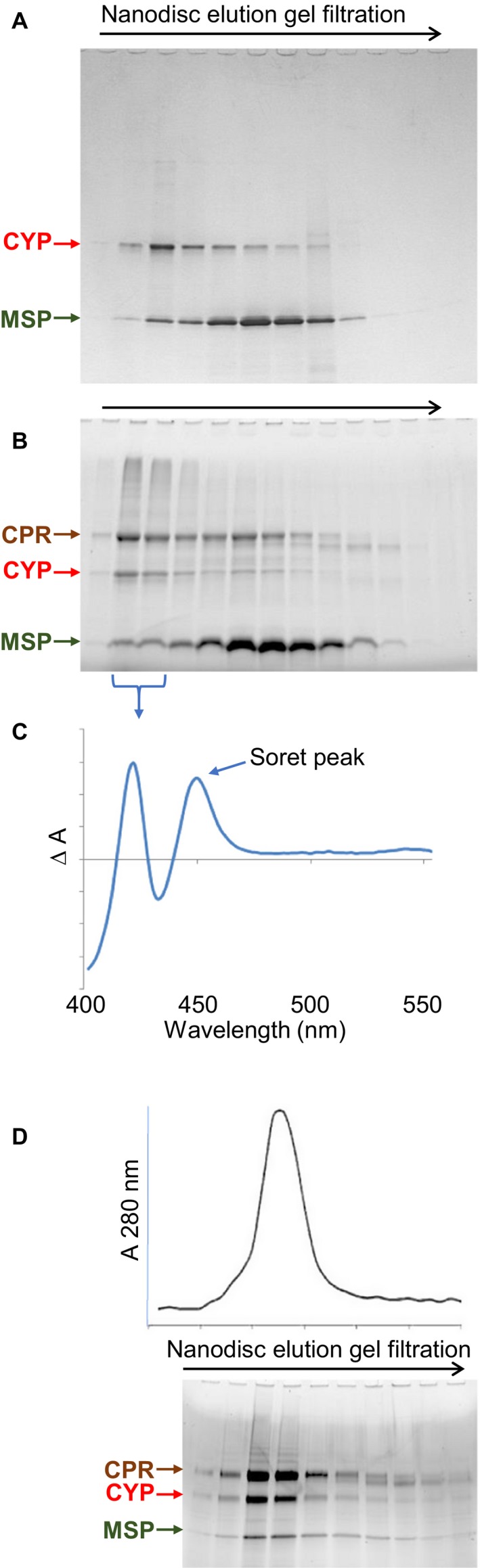
Separation and purification of CYP3A4 and CPR‐CYP3A4 nanodisc assemblies. (A) Separation of CYP3A4‐nanodisc assembly mixture by gel filtration. (B) Separation of CPR‐CYP3A4‐nanodisc assembly mixture by gel filtration. (C) Carbon monoxyferrous bound/unbound difference spectrum of the CYP3A4‐containing samples indicated. (D) Gel filtration separation of CPR‐CYP3A4‐nanodiscs after Ni‐affinity purification (using the His‐tag of CYP3A4, having first removed the His‐tag from MSP). The 280 nm absorbance profile was aligned to samples resolved by electrophoresis. CPR, CYP3A4 (CYP) and MSP1E3D1 (MSP) resolved by SDS/PAGE are indicated.

CYP3A4‐loaded nanodiscs, and doubly loaded nanodiscs containing both CPR and CYP3A4, were assembled by similar methods. Purification was achieved by a combination of gel filtration and enrichment by nickel affinity, making use of the C‐terminal His‐tag of the CYP3A4, having first removed the His‐tag from the MSP by cleavage with tobacco etch virus protease. Doubly loaded nanodiscs were assembled after first preincubating CPR and CYP3A4 together at a 2 : 1 molar ratio prior to mixing with the MSP and lipid. This possibly facilitated complex formation between the two proteins, and in our hands tended to result in final ratios of CPR to CYP3A4 of about 1 : 1 in the purified assembled nanodiscs. Figure 2 shows protein extracted from assembled nanodisc mixtures separated according to particle size by gel filtration, for both CYP3A4‐nanodiscs (Fig. 2A) and CPR‐CYP3A4‐nanodiscs (Fig. 2B). Figure 2C shows the carbon monoxyferrous bound/unbound difference spectrum of nanodisc fractions containing CYP3A4 (those indicated in Fig. 2B), of which the characteristic prominent Soret peak at 450 nm is an indication of enzymatically active CYP 47. Figure 2D shows how, following enrichment by nickel‐affinity, CPR‐CYP3A4‐nanodiscs resolve as a single homogeneous peak by gel filtration containing all three constituent proteins: CPR, CYP3A4 and MSP.

**Figure 3:**
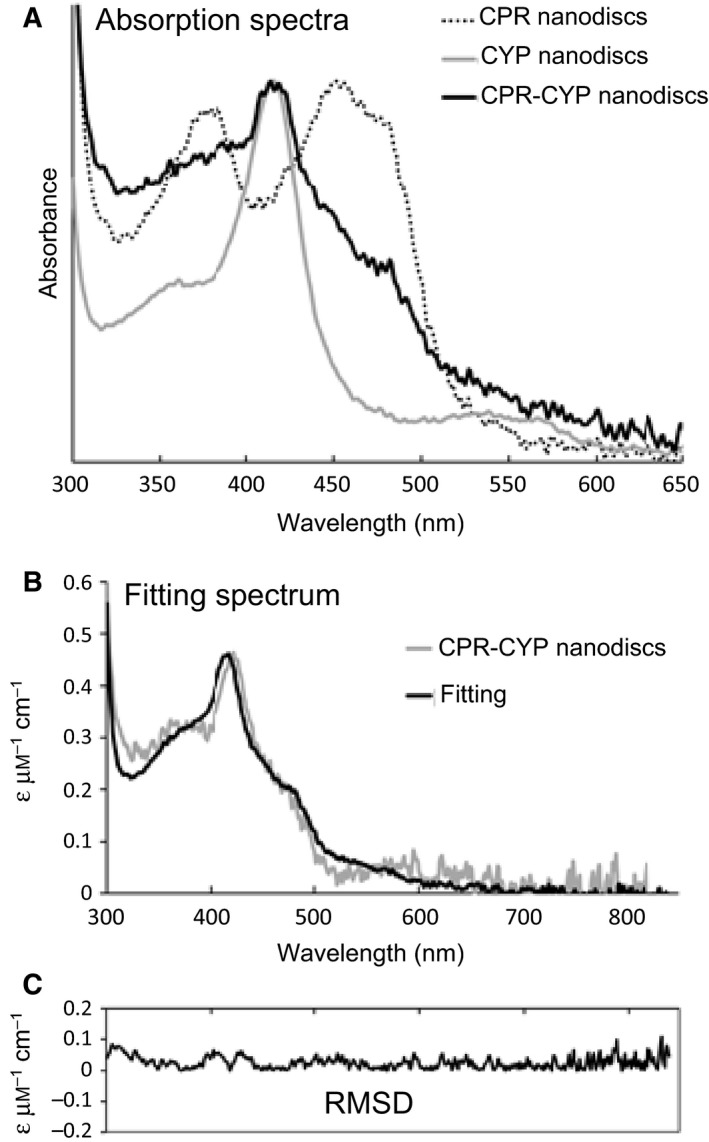
Determination of the CPR/CYP3A4 ratio in CPR‐CYP3A4‐nanodiscs. (A) Absorption spectra of CPR‐nanodiscs, CYP3A4‐nanodiscs and CPR‐CYP3A4 nanodiscs. (B) Fitting spectrum for the convolution analysis. Singly incorporated nanodisc spectra were used as reference. Molar extinction coefficients at the peaks of maximum absorption (CPR, 0.022 μm ^−1^·cm^−1^ and CYP3A4, 0.10 μm ^−1^·cm^−1^) were used to calculate the concentrations and molar ratio of the two enzymes in the doubly loaded nanodiscs. (C) Route‐mean‐square‐deviations (RMSD) for the fitting over the wavelength range. Mean RMSD was approximately 0.025.

The molar ratio of CPR to CYP3A4 in the doubly loaded nanodisc preparations was determined spectroscopically. A convolution spectrum was constructed by the linear combination of the absorbance spectra of nanodiscs individually loaded with CPR or CYP3A4 (Fig. 3A). This convolution spectrum was then fitted to spectra of doubly loaded nanodiscs using least‐squares regression (Fig. 3B,C), with the relative amount of each individual (CPR and CYP34A) spectra fitted for in terms of the concentration of CPR and CYP34A. Extinction coefficients of 0.022 μm ^−1^·cm^−1^ (CPR FMN maxima at ~ 450 nm) and 0.10 μm ^−1^·cm^−1^ (CYP34A Soret maxima) were used to determine individual protein concentrations and the preparations of doubly loaded nanodiscs used for kinetic analyses all had close to equimolar ratios of CPR to CYP3A4 (Table 1, cells 7A, 8A, 9A).

**Table 1:** Kinetic parameters. Det, detergent solubilized; ΔN, N‐terminally truncated (lacking membrane anchor); ND, nanodiscs; Mix, phospholipid mixture to mimic ER; Liv, liver microsomal lipid

	Enzyme	Enzyme form	Lipid type	A	B	C	D	E	F	G	H
				Molar ratio CPR : CYP	*k*_cat_ (s^−1^)	*K*_M_ (μm)	*k*_cat_/*K*_M_ (μm ^−1^·s^−1^)	*k*_cat_ (s^−1^)	*K*_M_ (μm)	*k*_cat_/*K*_M_ (μm ^−1^·s^−1^)	
					Electron donor			Electron acceptor			Hill coefficient
					*NADPH*			*Cytochrome c*			
1	CPR	Det	(–)		21.3 ± 6.4	10.0 ± 0.9	2.2 ± 0.8	29.9 ± 2.2	7.4 ± 2.4	4.6 ± 1.8	
2		ΔN	(–)		62.8 ± 2.7	11.8 ± 1.5	5.4 ± 0.9	78.2 ± 2.3	18.5 ± 0.9	4.2 ± 0.3	
3		ND	POPC		15.3 ± 0.3	5.4 ± 0.6	2.9 ± 0.4	15.7 ± 3.7	19.6 ± 6.2	0.96 ± 0.49	
4		ND	Liv		28.0 ± 0.5	5.2 ± 0.5	5.4 ± 0.6	46.2 ± 2.5	23.9 ± 2.8	1.97 ± 0.34	
					Cumene hydroperoxide			*7‐BQ*			
5	CYP	Det	(–)					0.18 ± 0.01	93 ± 13	1.9E‐3 ± 0.4E‐3	
6		ND	POPC					0.28 ± 0.03	82 ± 15	3.4E‐3 ± 1.0E‐3	
					*NADPH*			*7‐BQ*			
7	CPR+CYP	ND	POPC	0.8	110 ± 7	41 ± 6	2.8 ± 0.6	5.4 ± 0.2	54 ± 3	0.10 ± 0.01	2.1 ± 0.2
8		ND	Mix	1.1	32 ± 1	15 ± 2	2.2 ± 0.4	4.5 ± 0.2	37 ± 2	0.12 ± 0.01	1.8 ± 0.1
9		ND	Liv	1.0	179 ± 4	11 ± 1	16.4 ± 1.9	24.7 ± 0.5	29 ± 1	0.85 ± 0.05	2.1 ± 0.1

**Figure 4:**
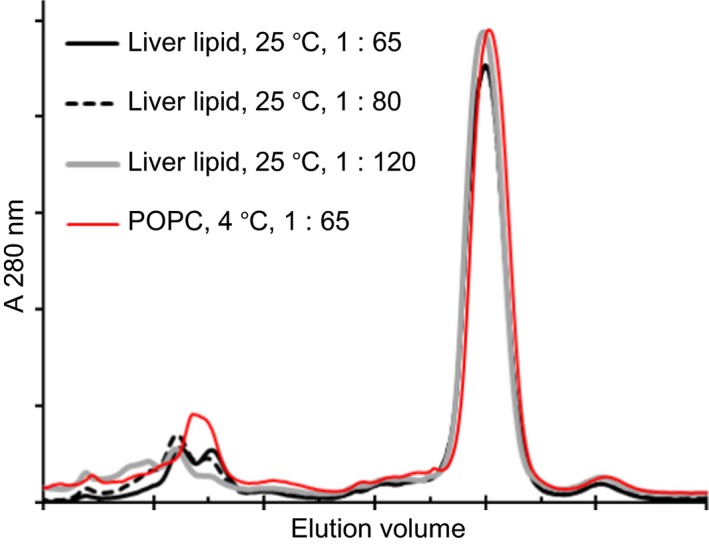
Size homogeneity of nanodiscs prepared with liver lipid compared with POPC. Identical gel filtration absorbance profiles showing homogeneity in size of nanodiscs prepared with varying liver lipid concentrations at 25 °C compared with POPC at 4 °C. MSP/lipid molar ratios are represented assuming a mean molecular mass of liver phospholipid of 760. Gel filtration separation was through Superdex 200.

In this study, we assembled nanodiscs not only with POPC but also with a crude preparation of natural phospholipid extracted directly from bovine liver microsomes (‘liver lipid’), and also a mixture of component phospholipids comprising the main constituents of liver ER, mixed in proportions approximately to match the natural composition of ER (‘mixed lipid’). The temperature of nanodisc assembly is recommended to correspond to the melting temperature of the lipid used 45; for liver and mixed lipids, we expected this to be 20 °C or above. A series of trials were performed (with no inserted membrane protein) to compare different ratios of lipid to MSP at different temperatures. The efficacy of nanodisc assembly was assessed by native gel electrophoresis and by gel filtration. Clear differences were not readily discernible by native gel electrophoresis, but the best resolved nanodisc complexes tended to form with liver lipid at 20 °C rather than at 4 °C or 37 °C, and at mass ratios of MSP to lipid similar to those used for assembly of nanodiscs with POPC at 4 °C (data not shown). Gel filtration profiles of nickel affinity‐enriched nanodiscs assembled at three different ratios of MSP to liver lipid at 20 °C were indistinguishable from the profile of nanodiscs assembled with POPC at 4 °C (Fig. 4). About 20 °C was therefore chosen as the standard temperature for the assembly of liver and mixed lipid nanodisc preparations, using the same lipid mass concentration as for POPC.

### CPR in liver lipid nanodiscs has enhanced activity with cytochrome *c* compared with CPR in POPC nanodiscs

**Figure 5:**
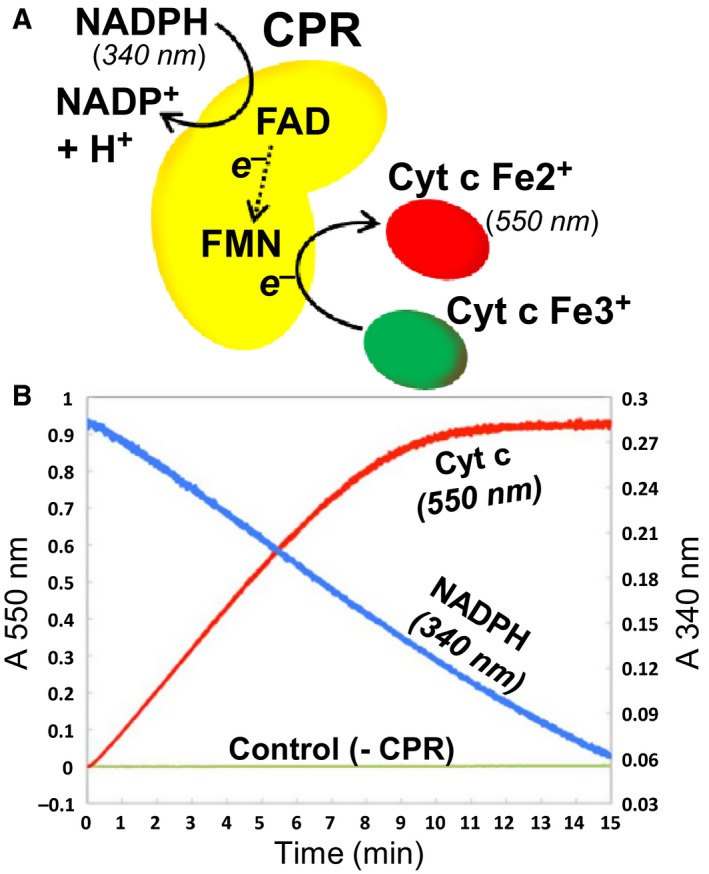
Assay of NADPH‐dependent cytochrome *c* reduction by CPR. Activity of CPR in nanodiscs was assayed spectroscopically by measuring both the consumption of NADPH, by decrease in 550 nm absorbance, and the reduction of cytochrome *c*, by increase in 340 nm absorbance, as shown schematically (A) or with time (B). The control trace lacking CPR includes NADPH and cytochrome *c*.

We sought to analyse the activity of the CPR‐CYP3A4 system by comparing the steady‐state kinetic parameters of the enzyme components, both singly and in combination, and in membrane‐integrated and in soluble form, in order to understand the significance of membrane integration and the influence of membrane composition. The results of the kinetic analyses are compiled in Table 1. A variety of assays were applied to compare the activities. First CPR was investigated alone, that is in the absence of its redox partner CYP3A4. *In vivo*, CPR serves as electron donor to CYPs. *In vitro,* however, it can also catalyse the reduction of cytochrome *c* from the reduced FMN domain of CPR. This reaction was assayed by two methods. First, the rate of formation of reduced cytochrome *c* was determined by measuring the increase in absorption at 550 nm. Second, the rate of oxidation of NADPH was determined by measuring the decrease in absorption at 340 nm. Thus, steady‐state kinetic parameters for the reaction were determined both with respect to cytochrome *c* reduction and NADPH oxidation (Fig. 5). Furthermore, CPR activity was assayed in four different forms: (a) the full‐length protein solubilized in detergent; (b) a truncated form of the protein (ΔN‐CPR) lacking sixty N‐terminal amino acids comprising the MAD; (c) the full‐length protein embedded in nanodisc membranes composed of POPC; and (d) the full‐length protein embedded in nanodisc membranes composed of liver lipid. Initial reaction velocities were fitted to the Michaelis–Menten equation and the measured parameters for the different forms of CPR are listed in Table 1, rows 1–4; those determined with respect to NADPH consumption are in columns B, C and D, and those determined with respect to cytochrome *c* reduction are in columns E, F and G.

Human CPR is membrane‐bound in its native state. No enhancement of CPR activity due to membrane‐binding was observed, as indicated by the catalytic efficiencies (*k*_cat_/*K*_M_) of the membrane‐bound forms, which are similar to, or less than, those of the soluble forms of CPR (Table 1, columns D and G, rows 1–4]. Cytochrome *c* is a soluble protein and membrane integration of CPR might be expected to have little effect on, or even to impede, the interaction of cytochrome *c* with CPR. The aqueously soluble ΔN‐CPR (lacking the MAD) exhibited the highest *k*_cat_ values (Table 1, cells 2B, 2E), although its catalytic efficiency was less dissimilar to the other forms (cells 2D, 2G). Upon comparing the effects of membrane composition on the activity of CPR in nanodiscs, consistent differences were observed: according to both assay methods, the catalytic efficiency of CPR in liver lipid nanodiscs was approximately twofold higher than in POPC nanodiscs. This is reflected in higher *k*_cat_ values in the liver lipid nanodiscs compared with POPC nanodiscs; the *K*_M_ values in the two different membrane compositions are equal (Table 1, columns B‐G, rows 3, 4). Thus, CPR in membranes composed of liver microsomal lipid has enhanced activity compared with CPR in membranes composed only of POPC.

### Membrane incorporation enhances O‐debenzylation of 7‐Benzyloxyquinoline by CYP3A4

CYP3A4 catalyses the O‐debenzylation of 7‐BQ to yield a fluorescent product, 7‐HQ, which can be quantitated by measuring emission at 515 nm upon excitation at 400 nm. In order to establish the assay, cumene hydroperoxide was used as an electron donor. The steady‐state kinetic parameters were then determined for the O‐debenzylation of 7‐BQ by both CYP3A4 solubilized in detergent, and by CYP3A4 incorporated in POPC nanodiscs (Table 1, columns E, F, rows 5, 6). Although the *K*_M_ values for the two forms of the protein were equal, the *k*_cat_ for CYP3A4 in the POPC nanodiscs was 50% higher than for CYP3A4 solubilized in detergent. Thus, incorporation of CYP3A4 into POPC membranes in nanodiscs appeared to confer some enhancement of activity over the detergent‐solubilized form. The reaction rates of CYP3A4 with cumene hydroperoxide as the electron donor were much lower than the rates with NADPH and CPR. For this reason, the comparison with liver lipid nanodiscs was not made, as it was considered to be of less physiological relevance.

### Cointegration of CPR and CYP3A4 in the same nanodiscs is essential for NADPH‐dependent CYP3A4 substrate conversion

**Figure 6:**
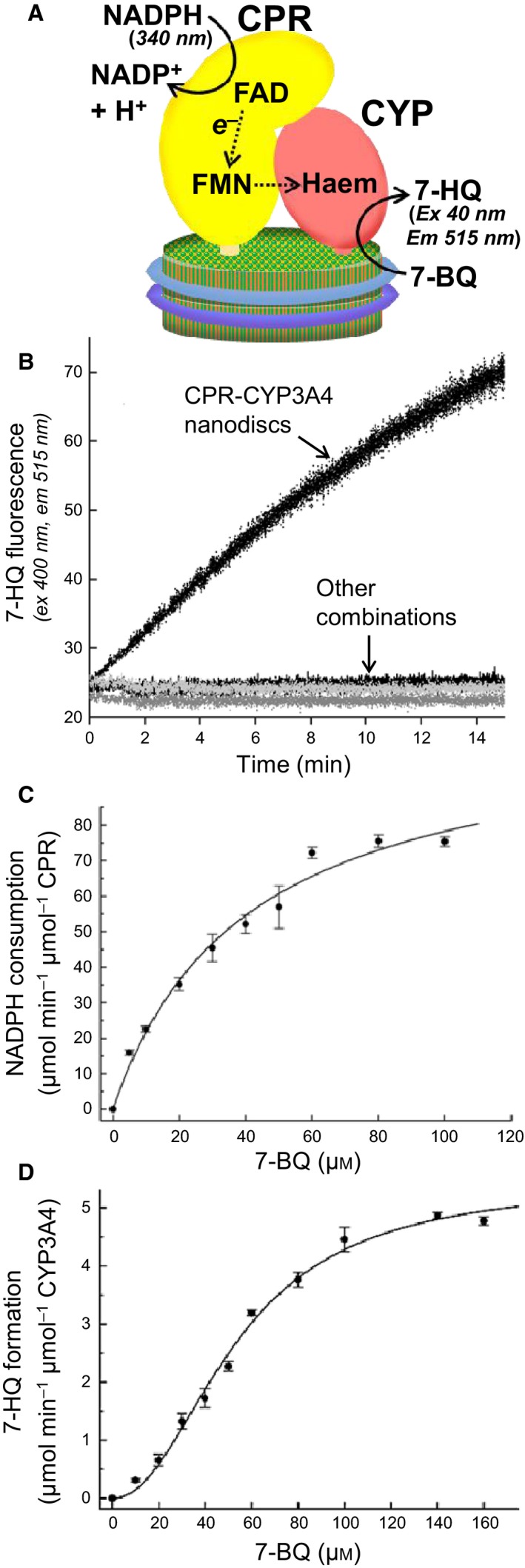
Catalytic conversion of 7‐BQ by CPR‐CYP3A4‐nanodiscs with NADPH as electron donor. (A) Activity of the coupled CPR‐CYP3A4 complex in nanodiscs was assayed both by measuring product formation (increase in 7‐HQ fluorescence emission at 400 nm), and consumption of NADPH (decrease in 550 nm absorbance). (B) NADPH‐dependent conversion of 7‐BQ by CPR‐CYP3A4‐nanodiscs. Only preassembled CPR‐CYP3A4‐nanodiscs showed activity. CPR‐nanodiscs alone, or CYP3A4‐nanodiscs alone, and buffer solution alone (all shown in this graph as ‘Other combination’) showed no activity. Other combinations also tested, but not shown in this graph, were equimolar mixtures of CYP3A4‐nanodiscs with CPR‐nanodiscs, CYP3A4‐nanodiscs with full‐length CPR and CYP3A4‐nanodiscs with ΔN‐CPR, all of which also gave negative ‘base‐line’ traces. Reactions contained 50 mm 7‐BQ and were initiated by addition 150 mm NADPH. (C) Steady‐state kinetic analysis of CPR‐CYP3A4‐nanodiscs with respect to NADPH consumption: initial reaction velocities plotted against substrate concentration best fitted the Michaelis–Menten equation. Error bars are standard deviation (*n* = 3). (D) Steady‐state kinetic analysis of CPR‐CYP3A4‐nanodiscs with respect to 7‐HQ formation: initial reaction velocities plotted against substrate concentration best fitted the Hill equation. Error bars are standard deviation (*n* = 3).

Cytochrome P450 reductase is the natural electron donor for CYP3A4‐mediated reactions, and both proteins in their natural state are membrane‐bound in the ER. We sought to study the coupled function of CPR and CYP3A4 and investigate the influence of membrane integration. To this aim we combined various forms of the two proteins to study the effects on O‐debenzylation of 7‐BQ. The only combination of CPR with CYP3A4 that supported conversion of 7‐BQ upon addition of NADPH was when both proteins were incorporated together within the same nanodisc (Fig. 6A,B). No combination involving any of the (membrane free) soluble forms of the proteins supported 7‐BQ conversion. Furthermore, no conversion was observed when separate preparations of CPR‐nanodiscs and CYP3A4‐nanodiscs were mixed together. Coincorporation of CPR and CYP3A4 within the same nanodisc phospholipid membrane, therefore, appears to be essential for the NADPH‐dependent O‐debenzylation activity.

### O‐debenzylation of 7‐BQ by CPR‐CYP3A4 in nanodiscs exhibits cooperativity with respect to substrate concentration

Having confirmed that 7‐BQ could serve as a substrate in an NADPH‐dependent manner for CYP3A4 in combination with CPR in nanodiscs (Fig. 6B), the steady‐state kinetic parameters of the reaction were determined both with respect to product formation rate, determined by measuring 7‐HQ fluorescence, and with respect to NADPH depletion rate, determined by measuring NADPH absorbance (illustrated schematically in Fig. 6A). When initial rates from the reaction curves were plotted against substrate concentration, the curve for NADPH consumption best fitted the Michaelis–Menten equation (Fig. 6C), whereas the curve for product formation best fitted the Hill equation (Fig. 6D). The latter indicated a cooperative effect on reaction rate due to binding of the substrate. The Hill coefficients for 7‐BQ O‐debenzylation by the coupled CPR‐CYP3A4 system in the three different membrane compositions had similar values; all had a value close to 2, indicating positive cooperativity of substrate binding (Table 1, cells 7H, 8H, 9H). Membrane composition therefore had no effect on the extent of the positive cooperativity of substrate binding.

### Liver microsomal lipid enhances both turnover and catalytic efficiency of the coupled CPR‐CYP3A4 enzyme pair in nanodiscs

The effects of varying membrane composition on the steady‐state kinetic parameters of the CPR‐CYP3A4 system in nanodiscs was investigated. CPR and CYP3A4 were coincorporated into nanodiscs of three different membrane compositions: (a) POPC; (b) ‘mixed lipid’, consisting (by % mass) of 62% PC, 20% phosphatidylethanolamine (PE), 10% phosphatidylinositol (PI), 5% sphingomyelin (SM), 2% phosphatidylserine (PS) and 1% phosphatyidic acid (PA), comprised to resemble the phospholipid composition of the ER; and (c) ‘liver lipid’, phospholipid extracted directly from bovine liver microsomes. The kinetic parameters are listed in Table 1, columns B–G, rows 7–9. In each case, the ratio of CPR to CYP3A4 in the nanodisc preparations, determined by deconvolution of absorbance spectra, was close to unity (Table 1, cells 7A, 8A, 9A).

The most obvious trend, on comparing these parameters is the enhancement of the coupled enzyme activity in the liver lipid nanodiscs, compared both to the nanodiscs composed of the mixed lipid, and to those composed of POPC. Catalytic efficiency in the liver lipid nanodiscs, as determined both by NADPH consumption and by 7‐BQ O‐debenzylation, is at least sixfold higher than in nanodiscs composed of the other lipids (Table 1, columns D, G, rows 7, 8, 9). This is a function both of higher *k*_cat_ and of lower *K*_M_ in the liver lipid nanodiscs (columns B, C, E, F, rows 7, 8, 9). Thus, not only does liver lipid enhance the activity of CPR alone in nanodiscs but it also enhances the activity of the coupled enzyme system, and to a greater extent than CPR alone. Differences between the mixed lipid and POPC were less apparent: a clear trend of increasing *K*_M_ from liver lipid => mixed lipid => POPC is apparent by both assay methods (columns C, F, rows 7, 8, 9), but there was little difference in catalytic efficiency between the mixed lipid nanodiscs and the POPC nanodiscs (columns D, G, rows 7, 8).

### CPR‐CYP3A4 decoupling occurs in nanodiscs, but is greater in POPC membranes than in liver or mixed lipid membranes

On comparing NADPH consumption with 7‐BQ O‐debenzylation of the coupled CPR‐CYP3A4 system, it is apparent that there is a significant discrepancy: NADPH consumption is several fold higher than 7‐BQ O‐debenzylation, in all three membrane compositions (Table 1, compare cells 7B, 8B, 9B with 7E, 8E, 9E). The discrepancy is highest with the POPC membranes, for which *k*_cat_ with respect to NADPH consumption is 20‐fold greater than with respect to 7‐BQ O‐debenzylation. With the mixed lipid and the liver lipid membranes, *k*_cat_ with respect to NADPH consumption is 7‐ and 11‐fold greater, respectively, than for 7‐BQ O‐debenzylation. These discrepancies suggest a degree of inefficiency or decoupling in the transfer of electrons from NADPH via CPR to CYP3A4, and that the decoupling is greater in the POPC membranes than in either the mixed lipid or liver lipid membranes.

### Superoxide production accompanies CPR‐CYP3A4 decoupling in nanodiscs

**Figure 7:**
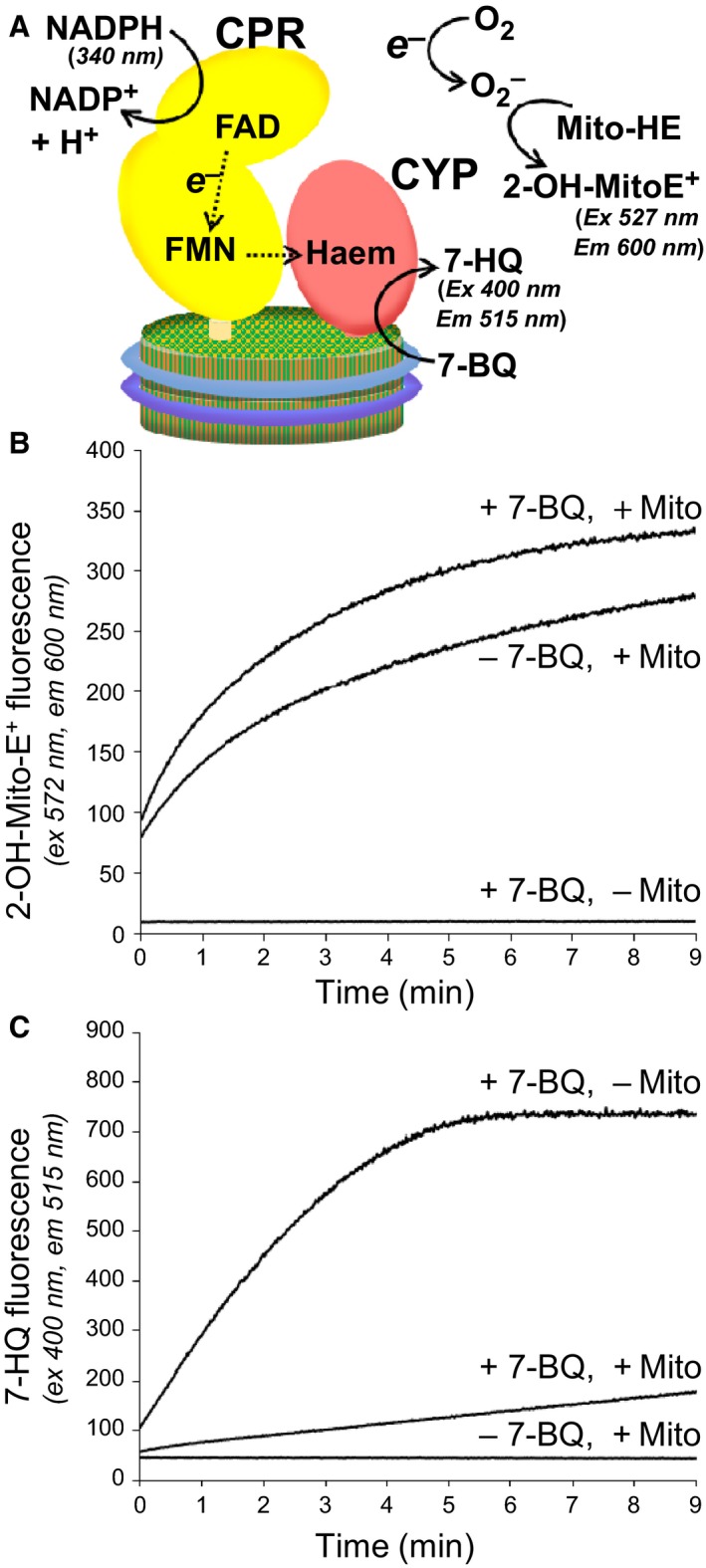
Measurement of NADPH‐dependent superoxide production by CPR‐CYP3A4‐nanodiscs. (A) NADPH‐dependent superoxide production from the coupled CPR‐CYP3A4 complex in nanodiscs was detected fluorimetrically upon conversion of the superoxide reporter Mito‐HE to 2‐OH‐Mito‐E^+^. Excitation and emission wavelengths are indicated. (B) Fluorescence monitoring of 2‐OH‐Mito‐E^+^ production with time upon addition of NADPH in the presence or absence of the CYP3A4 substrate 7‐BQ, and in the presence or absence of Mito‐HE (Mito). (C) Fluorescence monitoring of 7‐HQ production with time upon addition of NADPH in the presence or absence of 7‐BQ, and in the presence or absence of Mito‐HE. Concentrations of 7‐BQ and Mito‐HE in the starting mixtures were 100 and 10 μm respectively. Reactions were initiated by addition of 150 μm NADPH.

It has long been known that decoupling of electron transfer can occur in CPR‐CYP systems 48. Decoupling in the presence of molecular oxygen gives rise to the formation of reactive oxygen species, notably superoxide ($O_{2}^{-}$). In order to demonstrate the production of superoxide during 7‐BQ O‐debenzylation by the CPR‐CYP3A4 system we used the hydroethidine‐derived fluorescent dye Mito‐HE. Reaction of Mito‐HE with superoxide forms a specific red‐fluorescent 2‐hydroxyethidium product (2‐OH‐Mito‐E^−^), which serves as an indicator of superoxide 49, 50 (Fig. 7A).

Mito‐HE was added to the CPR‐CYP3A4 nanodisc reaction mixture, with 7‐BQ as substrate. The reaction was initiated by addition of NADPH, and the change in red fluorescence was monitored by measuring 600 nm emission upon 527 nm excitation. A marked increase in red fluorescence was initiated by the addition of NADPH, which continued throughout the course of the reaction, indicating the generation of superoxide (Fig. 7B). When Mito‐HE was omitted from the reaction, no increase in red fluorescence was observed, as expected. In the absence of 7‐BQ as substrate, when Mito‐HE was included, an increase in red fluorescence was also observed, indicating that superoxide was generated by the system even in the absence of substrate; this increase, however, was less than when substrate was included (Fig. 7B). 7‐HQ product formation was monitored simultaneously by measuring 515 nm emission upon 400 nm excitation. In the absence of Mito‐HE, 7‐HQ formation proceeded as expected from previous kinetic measurements. However, in the presence of Mito‐HE, the rate of 7‐HQ formation was significantly reduced (Fig. 7C). This indicated that Mito‐HE had an inhibitory effect on the reaction; the reaction still proceeded, but at a reduced rate.

Thus, in the presence of Mito‐HE, the induction of red fluorescence upon NADPH addition indicates that superoxide is produced by the CPR‐CYP3A4 system in the absence of substrate, but that superoxide production is significantly enhanced during substrate conversion. These results therefore suggest that electron ‘leakage’ occurs at multiple stages during electron transfer, both on transit between the flavin cofactors of CPR, and during the CYP3A4 reaction cycle. This is consistent with what is already known about uncoupling in the CPR‐CYP system, in that reactive oxygen species are known to be generated both in the presence and absence of the CYP substrate upon addition of NADPH 48.

## Discussion

We have used phospholipid bilayer nanodiscs to investigate the significance of membrane insertion and the influence of membrane composition with respect to the activity of human CPR and CYP3A4. It has long been appreciated that trypsin‐solubilized CPR isolated from liver microsomes lacks a portion of the N terminus required to tether the protein to microsomal membranes, and that this soluble form while retaining the capacity to catalyse NADPH‐dependent reduction of cytochrome *c*, is unable to couple effectively with CYP and support CYP mono‐oxygenase activity 11, 51. The detergent‐solubilized full‐length form of the protein could be incorporated spontaneously into microsomes or synthetic phospholipid vesicles, whereas the cleaved form could not 52. In assembling CPR into nanodiscs, by observing the colour conversion of MTT in native polyacrylamide gels, we have been able to confirm in a very clear and direct way the essential requirement for the N‐terminal domain in tethering CPR to the phospholipid membrane. The full‐length heterologously expressed CPR clearly became incorporated into nanodiscs, whereas the 60‐amino acid N‐terminally truncated form (ΔN‐CPR) did not (Fig. 1A).

Upon incorporation of CYP3A4 into nanodiscs, and having developed a convenient fluorimetric assay for the enzyme using 7‐BQ as substrate (initially using cumene hydroperoxide as electron donor), we tested which combinations of various forms of CPR with CYP3A4‐nanodiscs would support reconstitution of the coupled NADPH‐dependent activity. The only condition which supported reconstitution of the activity was when CPR and CYP3A4 were coincorporated in the same nanodiscs. No activity was detected when CPR‐nanodiscs and CYP3A4‐nanodiscs were assembled separately then mixed together, nor when either of the soluble forms of CPR (either the full‐length detergent‐solubilized or the ΔN‐CPR form) were mixed with the CYP3A4‐nanodiscs. The requirement for membrane insertion of CPR for reconstitution of the coupled activity has been previously inferred from, first, the requirement for an intact MAD on CPR 12, 51, 52, and second, the requirement for phospholipid in the reconstitution mixture 16. Taken together, our results are a direct demonstration of the requirement for coinsertion of CPR and CYP3A4 within the same phospholipid membrane for reconstitution of activity. Interestingly, yeast CPR lacking the N‐terminal MAD does support CYP‐dependent sterol biosynthesis, both *in vivo* and in a reconstituted assay 53. The explanation for this apparent qualitative difference between yeast and mammalian CPR is not known. It is possible that the N‐terminal MAD of mammalian CPR is required to interact directly with CYP as well as to serve as a membrane anchor 11. The requirement for similar interaction in yeast has not been demonstrated, and it may be that the residues required for this interaction are lacking from the yeast CPR N‐terminal domain.

In looking at the effect of membrane insertion on CYP3A4 activity, we found that turnover (*k*_cat_) was slightly enhanced by insertion into nanodiscs, compared with the nonmembrane‐bound detergent solubilized CYP3A4 (Table 1, cells 5E, 6E). This result is not unexpected. It is consistent with molecular dynamics studies that suggest that one face of the protein could lie well embedded within the membrane surface and thus facilitate the access of amphiphilic substrates, which have the potential to accumulate within the membrane 13, 17, 18, 19.

When the activities of various forms of CPR alone were compared, with an assay using cytochrome *c* as a substrate, ΔN‐CPR consistently showed the highest rates compared with the full‐length CPR, with respect to both NADPH consumption and cytochrome *c* reduction (Table 1, cells 2B, 2E). It has been reported previously that trypsin cleavage (which results in loss of the N‐terminal MAD 12) enhances the activity of mammalian CPR with respect to cytochrome *c* reduction by a factor of up to 1.25 51. Our data show rate enhancements (*k*_cat_) for the N‐terminally truncated CPR of from 1.7‐ to 5‐fold. It is not clear why the ΔN‐CPR should exhibit higher rates, but presumably loss of the MAD makes CPR more freely accessible to interact with cytochrome *c*, which, unlike the natural CYP redox partner of CPR, is small, not membrane‐bound and freely diffusible in aqueous medium. It is known that CPR adopts multiple conformations and that each will react differently with cytochrome *c* 54, 55. Truncation of the MAD in the ΔN‐CPR is expected to affect the conformational landscape of CPR.

When inserted into nanodiscs, the activity of CPR with cytochrome *c* was consistently higher in membranes composed of liver microsomal lipid compared with membranes composed of POPC, in terms of both the catalytic constant (*k*_cat_) and the catalytic efficiency (*k*_cat_/*K*_M_), and according to both NADPH consumption and cytochrome *c* reduction (Table 1, rows 3, 4). As far as we are aware, this is the first report of enhanced activity of membrane‐bound CPR alone in liver lipid compared with PC. The redox potentials of CPR incorporated alone in nanodiscs have been compared in POPC membranes and in membranes composed of 50% POPC/POPS, and have been found to differ 37. The differences in potential between the various flavin states, it was proposed, were such as to promote more efficient electron transfer in the more anionic POPC/POPS mixture than in POPC alone. Whether the anionic phospholipid content of liver microsomal lipid could influence flavin redox potential so as to cause the enhanced CPR activity we observed in liver lipid nanodiscs is not known. The 50% POPC/POPS mixture is not physiologically relevant, and the total content of anionic phospholipid in liver microsomes is substantially less than 50% 56, 57. The mechanism by which CPR reaction rates are enhanced by liver lipid, therefore, remains to be determined.

Liver lipid substantially enhanced the coupled activity of CPR‐CYP3A4 in nanodiscs, determined using the 7‐BQ O‐debenzylation assay. Furthermore, the enhancement of the activity of the coupled system with respect to 7‐BQ O‐debenzylation was greater than the enhancement of the activity of CPR alone with respect to cytochrome *c* (Table 1, compare 4E/3E with 9E/7E, and 4G/3G with 9G/7G). With respect to NADPH consumption, the degree of enhancement was similar with regard to *k*_cat_ (Table 1, compare 4B/3B with 9B/7B) but markedly greater with regard to catalytic efficiency (compare 4D/3D with 9D/7D). This extra enhancement of the coupled enzyme activity suggests that we have detected an important mechanistic phenomenon relevant to the activity of the enzyme system in its natural state in the ER. It indicates that membranes composed of liver lipid have properties or contain components that are important for efficient catalysis by the coupled CPR‐CYP3A4 system, which are lacking from membranes composed purely of POPC. The phenomenon affects CPR alone, but it affects the coupled CPR‐CYP3A4 complex to a markedly greater extent. This enhancement by liver microsomal lipid is consistent with that previously observed for CPR‐CYP3A4 in lipid vesicles, using different substrates 32. Our results therefore confirm and expand the relevance of this previous study.

We sought to investigate what the special properties of the liver lipid membrane might be by performing a further comparison which involved incorporation of CPR‐CYP3A4 into nanodiscs composed of a mixture of purified phospholipids combined in proportions to reflect the natural composition of the ER (PC 62%, PE 20%, PI 10%, SM 5%, PS 2% and PA 1%). However, in this case there was no enhancement compared with POPC (Table 1, row 8). We conclude, therefore, that the properties of, or components within, natural liver lipid which enhance CPR‐CYP3A4 activity must be in addition to, or not directly related to, the combination of phospholipids in our ER‐mimic mixture. Natural liver microsomal lipid contains additional components other than those included in our mixture, some of which are known to influence enzyme activity. Cholesterol, for example, is known to have significant effects on the activities of a number of membrane proteins 58 and can constitute as much as 10% (by phosphorus content) of the lipid in rat liver microsomal preparations 56, 57. Also, the anionic phospholipid cardiolipin, which has been reported to have a specific effect in stimulating the activity of human CYP1B1 in a manner dependent on the protein having an intact N‐terminal MAD 24, was not included in our phospholipid mixture. Cardiolipin is a relatively minor phospholipid component of the ER (~ 1% 57) and the effects described with CYP1B1 were observed at unnaturally high concentrations. However, cardiolipin has been suggested to have a specific role as a cofactor in the regulation of the activity of the yeast cytochrome *bc*1 complex, in which it has been proposed to maintain the structural integrity of proton‐conducting channels 59. At present, however, the properties of liver microsomal lipid which enhance the activity of CPR‐CYP3A4 in nanodiscs remain unknown and must remain the focus of future study.

The CPR‐CYP3A4 activity in nanodiscs revealed a significant degree of decoupling. That is, NADPH was consumed at a rate higher than could be accounted for by substrate conversion (Table 1, compare 7B, 8B, 9B with 7E, 8E, 9E). This implied that the system was ‘leaking’ electrons and this leakage was expected to give rise to the generation of superoxide (illustrated schematically in Fig. 7A). It was possible to demonstrate the generation of superoxide using Mito‐HE, which upon reaction with superoxide is converted to a product (2‐OH‐Mito‐E^+^) with diagnostic fluorescent signal. Superoxide was not only generated during NADPH‐dependent CPR‐CYP3A4‐substrate conversion but also, although to a lesser extent, in the absence of substrate (Fig. 7B). Superoxide generation in the absence of substrate suggests electron leakage from the CPR flavin‐coupled electron transfer, as has been described previously 60, while the enhanced superoxide generation observed in the presence of substrate presumably represents the sum of leakage both from the CPR flavin‐coupled electron transfer and from the CYP3A4 reaction cycle 48. Addition of Mito‐HE to the coupled reaction mixture resulted in partial inhibition of 7‐BQ conversion (Fig. 7C). The cause of this is not understood, but possibly Mito‐HE competes with 7‐BQ as substrate for CYP3A4.

The extent of decoupling was affected by the composition of the nanodisc membranes. Compared with POPC, liver microsomal lipid not only enhanced CPR‐CYP3A4 activity but also tightened the coupling of electron transfer (Table 1, compare 9B/9E with 7B/7E). The mixed phospholipid (ER mimic) membrane also enhanced coupling, to the same extent as liver lipid (Table 1, 8B/8E), although unlike liver lipid it failed to enhance activity. This suggests that the membrane properties or components responsible for enhancing activity are not necessarily the same as those responsible for tightening redox coupling, since the mixed phospholipid appears to enhance one but not the other, whereas the liver microsomal lipid enhances both. Cardiolipin has been found to reduce the output of reactive oxygen species by human CYP2E1 in phospholipid vesicles in a manner dependent on an intact N‐terminal MAD 23. Also both cardiolipin and PA have similar MAD‐dependent effects in enhancing the activity of human CYP1B1 24. It is conceivable, therefore, that the PA in the mixed phospholipid membrane could affect coupling in our study. However, further detailed comparisons under carefully controlled conditions will be necessary to clarify such influences. By studying CPR‐CYP3A4 as a defined 1 : 1 coupled complex in nanodiscs, we have shown at least that such comparisons are feasible.

In conclusion, the properties of nanodiscs that enable the preparation of monodisperse particles of relatively even size and of defined composition have allowed us to demonstrate in a very direct way the importance of tethering CPR together with CYP3A4 within the same membrane in order to reconstitute NADPH‐dependent mono‐oxygenase activity. We have furthermore shown how natural liver lipid has properties which enhance this activity, compared with membranes of synthetic phospholipid composition. The use of nanodiscs has enabled us to define this phenomenon with precision, with greater control over the stoichiometry of the enzyme system. Our findings emphasize the importance of using natural lipid preparations for the detailed study of the activity of membrane proteins in order better to understand the regulatory effects imposed by the membrane environment.

## Experimental procedures

### Protein production and purification

All protein purification steps were performed at 4 °C or in ice slurry. Plasmids pMSP1D1 and pMSP1E3D1 for expression of MSPs were obtained from Addgene (www.addgene.org). MSPs were expressed and purified as described by Ritchie *et al*. 45, except that expression was induced in batch cultures of 0.5 L Terrific Broth (TB) in 2‐L flasks shaken at 30 °C and 200 r.p.m. for 5–6 h after induction.

Plasmid pPORh1 for expression of human CPR (derived by substitution of human for rat CPR cDNA in pOR263 61, 62) was kindly provided by Bettie Sue Masters, University of Texas Health Science Centre at San Antonio, TX, USA, who also provided a method on which the following procedure was based. CPR from pPORh1 in *Escherichia coli* has an N‐terminal *E. coli* outer membrane protein A (OmpA) leader to target the protein for periplasmic membrane insertion. A single colony of *E. coli* C41(DE3) transformed with pPORh1 was used to inoculate 5 mL ‘Lauria–Bertani’ broth (LB) broth containing 50 μg·mL^−1^ carbenicillin. After 5–6 h of growth at 37 °C, the culture was diluted in 250 mL TB containing 50 μg·mL^−1^ carbenicillin, and allowed to grow overnight at 37 °C. Six 2 L flasks, each containing 0.5 L TB, 50 μg·mL^−1^ carbenicillin, were each inoculated with 30 mL of the overnight culture and grown at 28 °C at 180 r.p.m. to an optical density of 0.8–1.0 (1 cm light‐path at 600 nm wavelength). The incubation temperature was then decreased to 21 °C for 30 min, 0.5 mm riboflavin (a precursor of the flavin cofactor) was added, CPR synthesis was induced by addition of IPTG at 0.4 mm final concentration, and the culture was grown for a further 24–30 h at 21 °C. Cells were harvested by centrifugation and stored at −80 °C.

Thawed cells were suspended in two volumes of lysis buffer (100 mm Tris‐HCl, pH 7.6, 1 mm EDTA, 10% glycerol, 1 mm DTT), the suspension was stirred until homogeneous, lysozyme and DNase I were added, each to final concentration 10 μg·mL^−1^, ‘Complete EDTA‐free’ protease inhibitor tablets (Roche Products Ltd, Welwyn Garden City, UK) were added (2 per 100 mL), and the suspension was stirred gently for a further 30 min. Cells were disrupted by sonication, coarse debris was removed by centrifugation [17 000 ***g*** (max), 20 min], then microsomal membranes were sedimented by further centrifugation [180 000 ***g*** (max), 1 h]. The membrane‐containing pellets were suspended to homogeneity in buffer C (50 mm Tris‐HCl, pH 7.7, 0.1 m EDTA, 10% glycerol, 0.05 mm DTT, 1% Triton X‐100) in an homogenizer with motor‐driven rotating Teflon pestle, two extra protease inhibitor tablets were added, and the homogenized suspension was stirred gently overnight to solubilize the CPR. The extract was then clarified by a second centrifugation step at 180 000 ***g*** (max) for 1 h.

Cytochrome P450 reductase was purified from the clarified supernate by affinity chromatography on 2′,5′‐ADP Sepharose 4B, as follows. Protein was applied to a 15‐mL bed‐volume of resin pre‐equilibrated in buffer C, washed further with buffer C, then with 100 mL of 5 mm adenosine in buffer C. A two‐stage gradient of increasing concentration of 2′(3′)‐adenosine monophosphate (mixed isomers) in buffer C was applied to elute the CPR: a first stage of 0–0.5 mm (100 mL), followed by a second stage of 0.5–5.0 mm (100 mL). The two‐stage elution effectively separates the intact, full‐length CPR from a proteolytically cleaved form which lacks the MAD. After excision of the appropriate band from a polyacrylamide gel, the full‐length protein was sent for N‐terminal amino acid sequence determination (Alta Biosciences Ltd, University of Birmingham, Birmingham, UK) and found to have the N terminus Gly‐Iso‐Pro‐Gly‐Ser, which corresponds to the last five amino acids of the OmpA leader; this corresponds to the same N terminus mapped for other OmpA fusions 63. Fractions containing full‐length CPR were pooled then concentrated with a 30‐kDa molecular weight exclusion filter. CPR concentration was determined according to the bound flavin content by absorbance measurement at 454 nm, using an extinction coefficient ε = 21.4 mm ^−1^·cm^−1^ 64. Purified CPR in buffer containing 10% glycerol (v/v) was frozen in liquid nitrogen and stored at −80 °C.

A His‐tagged, soluble form of human CPR, lacking the 60 amino acids from the N terminus comprising the MAD (ΔN‐CPR), was expressed and purified as described previously 65.

Plasmid pB84 for expression of C‐terminally His‐tagged human CYP3A4 was kindly provided by R. Wolf, University of Dundee, UK, who also provided a method on which the following purification procedure was based. pB84 is derived from a plasmid for membrane protein expression in *E. coli*, derived from pCW as previously described 66. Like CPR, CYP3A4 was expressed with an OmpA leader sequence as periplasmic membrane targeting signal, which is cleaved off as part of the targeting process, leaving the protein associated with the membrane by its own N‐terminal MAD. *E. coli* strain K12 JM109 was transformed with pB84 and a single colony was used to inoculate 50 mL LB culture containing 50 μg·mL^−1^ carbenicillin and grown at 37 °C overnight. Several 2‐L flasks containing 0.5 L TB, 50 μg·mL^−1^ carbenicillin, were inoculated each with 6 mL of the overnight culture and grown at 37 °C, 180 r.p.m., to optical density 0.8–1.0 (1 cm light‐path at 600 nm). The incubation temperature was then decreased to 28 °C, 1 mm 5‐aminolevulinic acid (a precursor to the haem cofactor) was added, then CYP synthesis was induced by addition of 1 mm IPTG, and culture was continued for a further 20–24 h at 28 °C. Cells were harvested by centrifugation and stored at −80 °C.

Thawed cells were suspended in up to five volumes of lysis buffer: 50 mm potassium phosphate, 500 mm potassium chloride, pH 7.4, 20% glycerol. Lysozyme and DNase I were added each to 10 μg·mL^−1^ final concentration, the suspension was stirred in ice until homogeneous, protease inhibitors were added (as for CPR), and cells were disrupted by sonication. CHAPS detergent was added to 10 mm final concentration and the lysate was stirred gently overnight. Debris was removed by centrifugation [48 000 ***g*** (max), 50 min], then the supernate was applied to a Ni‐nitrilotriacetic acid agarose column (25 mL resin), pre‐equilibrated with 50 mm potassium phosphate, 0.5 m potassium chloride, pH 7.4, 20% glycerol. The column was washed with five bed‐volumes of the same buffer, then sequentially with the same buffer containing: (a) 5 mm CHAPS (b) 30 mm imidazole; and (c) 50 mm imidazole. CYP3A4 was then eluted in 0.4 m imidazole in 40 mm Tris‐HCl buffer. Fractions containing CYP3A4 were pooled and dialysed against 100 mm potassium phosphate, 0.1 mm DTT, 0.1 mm EDTA, 5 mm CHAPS, pH 7.4, 20% glycerol, then concentrated with a 30 kDa molecular weight exclusion filter. The concentration of haem in the purified CYP3A4 preparations after dialysis was determined by pyridine haemochromagen assay 67, 68. This corresponded well with the concentration determined by 420 nm absorption using an extinction coefficient of ε_420 nm_ = 100 mm ^−1^·cm^−1^. It was valid, therefore, to use the absorbance spectrum of the oxidized form of CYP3A4 in the calculation of CYP3A4/CPR ratios in the doubly loaded nanodisc preparations (see below).

### Phospholipids and phospholipid mixtures

Phospholipids (from Sigma‐Aldrich Ltd, Gillingham, UK or Generon Ltd, Slough, UK) were stored under nitrogen, either desiccated or dissolved in methanol/chloroform (2 : 1), in Teflon‐capped glass vials at −20 °C. Nanodiscs were assembled either with 2‐oleoyl‐1‐palmitoyl‐sn‐glycero‐3‐phosphocholine (POPC), or a mixture of phospholipids intended to mimic the composition of the mammalian ER, or with crude phospholipid extracted from bovine liver microsomes. The phospholipid mixture to mimic the ER was composed as follows (% mass): PC 62%, PE 20%, PI 10%, SM 5%, PS 2% and PA 1% 56, 57. Liver microsomal phospholipid was prepared as follows. Ox liver fresh from slaughter was chilled on ice, diced and homogenized in a blender with 2–3 volumes of 0.25 m sucrose, 0.1 m potassium phosphate, pH 7.4. Coarse debris was removed from the slurry by two sequential centrifugation steps of 15 min at 14 000 ***g*** (max), microsomes were then sedimented from the supernate by ultracentrifugation (140 000 ***g***, 1 h). The resulting deep red, glassy, gelatinous pellets were stored at −80 °C until required. Phospholipid was extracted by the method of Bligh and Dyer 69, whereby microsomes suspended in a minimal volume of buffer were homogenized in methanol and chloroform in such proportion that a miscible system was formed; dilution with chloroform and water then caused phase separation, with lipid retained in the chloroform phase and nonlipid in the aqueous phase. Lipid yield was estimated by weighing the residue from an aliquot dried under nitrogen and lyophilized under vacuum. Appropriate quantities of phospholipid were dispensed in methanol/chloroform, dried and dissolved in 0.1 m sodium cholate, 20 mm Tris‐HCl, pH 7.4, at 38 mg·mL^−1^ (equivalent to 50 mm POPC) immediately prior to requirement for nanodisc assembly.

### Assembly and purification of nanodiscs

Assembly of phospholipid bilayer nanodiscs was essential as previously described 42, 45. To constitute CPR into nanodiscs, CPR was mixed with the membrane scaffold protein MSP1D1 and cholate‐solubilized POPC at a molar ratio of 1 CPR: 65 MSP1D1: 130 POPC in a solution containing 10 mm POPC, 20 mm sodium cholate, 0.1 m NaCl, 20 mm Tris‐HCl, pH 7.4. For nanodiscs composed of liver microsome and mixed phospholipids, mass concentrations equivalent to POPC were used. After mixing by rotation for 1 h at 4 °C, assembly was initiated by addition of an approximately equal volume of Amberlite XAD‐2 beads (prewashed and equilibrated in buffer), and the total mixture was incubated on a roller for 20–24 h at 4 °C. Assembly of nanodiscs with liver microsome or mixed lipid was performed at 20 °C to account for the higher melting temperature of these lipid mixtures. Purification of the assembled nanodiscs was by Ni affinity to the His‐tag of the MSP. After removal of the Amberlite beads, the assembly mixture was applied to Ni‐nitrilotriacetic acid resin (or HisTrap HP column) pre‐equilibrated in 40 mm Tris‐HCl pH 7.4, 0.3 m NaCl, and washed successively with 10 and 50 mm imidazole, then eluted with 0.3 m imidazole in the same buffer. Purity of the eluted fractions was assessed by SDS/PAGE. Nanodiscs could also be enriched by gel filtration (Superdex 200, or Sephacryl S‐200 or 300, GE Healthcare Life Sciences, Little Chalfont, UK), and analysed by nondenaturing PAGE [precast TGX gels (Bio‐Rad Laboratories, Watford, UK) or 9% acrylamide (30 : 0.8 mono/bis) in 25 mm Tris‐glycine, pH 8.3, at 100 V]. Nanodisc‐containing fractions were pooled, dialysed against 20 mm Tris‐HCl, 0.1 m NaCl, pH 7.4, concentrated by filtration and stored at −80 °C.

The method to constitute CYP3A4 into nanodiscs was similar to that for CPR, but to purify CYP3A4‐nanodiscs, the His‐tag was first removed from the MSP by cleavage with tobacco etch virus protease, leaving the His‐tag on CYP3A4 alone available for affinity purification. Ni‐affinity purification was then performed as for CPR nanodiscs.

For simultaneous incorporation of both CPR and CYP3A4 into nanodiscs, the larger MSP1E3D1 was used, better to accommodate both proteins, the His‐tag first having been removed to allow affinity purification via the His‐tag attached to CYP3A4. CYP3A4 and CPR were premixed and incubated together for 30–60 min at room temperature to facilitate possible binding between the two proteins, then all components were mixed in a molar ratio of 0.1 CPR: 0.2 CYP3A4: 1 MSP1E3D1: 90 POPC: 180 cholate, as recommended 42. Assembly then proceeded as described above for CPR‐nanodiscs. Following Ni‐affinity enrichment (via the His‐tag attached to CYP3A4), CPR remained associated with the nanodisc at approximately equimolar ratio with CYP3A4; gel filtration was then sufficient to give an homogeneous preparation of CPR‐CYP3A4‐nanodiscs.

CPR : CYP3A4 ratios were determined by absorbance spectroscopy using a NanoDrop 2000 UV‐vis spectrophotometer (Thermo Fisher Scientific, Waltham, MA, USA), with fivefold repetition of readings. The two singly loaded nanodisc spectra in Fig. 3A were used as basis spectra and a convolution spectrum was created by the linear combination of these spectra. Fitting of the convolution spectrum to the doubly loaded nanodisc spectra allowed the concentration and molar ratios of the two proteins in the doubly loaded nanodisc preparations to be estimated. The fitting method was based on an algorithm to minimize root‐mean‐square deviation between the experimental and convolution spectra. The fitted spectra matched the scanned spectra well, with mean root‐mean‐square deviation approximating to 0.025 across the wavelength range. Preparations with CPR : CYP3A4 ratios close to 1 : 1 were used for kinetic analyses.

### Enzymic reactions

#### Detection of CPR activity in native gels by reduction of thiazolyl blue tetrazolium bromide

Following electrophoresis, gels were rinsed twice in water, soaked in a minimal volume of freshly mixed 0.5 mm NADPH, 1 mm MTT, 0.1 m potassium phosphate, pH 7.6, incubated at 37 °C for up to 30 min to allow colour development, then rinsed further in water and photographed.

#### NADPH‐dependent cytochrome *c* reduction by CPR

Cytochrome P450 reductase was first treated with potassium ferricyanide to oxidize the flavin cofactors, then repurified by passage through a desalting column. Reactions were performed in disposable plastic cuvettes in a UV‐visible spectrometer within an anaerobic glovebox. CPR and cytochrome *c* were mixed in 0.3 m potassium phosphate, pH 7.4, at 25 °C, in a range of concentrations, and reactions were initiated by the addition of NADPH. Change in absorbance was recorded either at 550 nm to measure cytochrome *c* reduction, or 340 nm to measure NADPH consumption. Initial velocities were fitted to the Michaelis–Menten equation.

#### Fluorescence assay of O‐debenzylation of 7‐benzyloxyquinoline by CYP3A4

O‐debenzylation of 7‐BQ by CYP3A4 gives rise to the fluorescent product, 7‐HQ, which can be detected by measuring emission at 515 nm upon excitation at 400 nm. Enzymes or nanodisc preparations were mixed in 0.1 m potassium phosphate, 7.5 mm magnesium chloride, pH 7.4, with 7‐BQ (0.1–200 μm) in fluorescence quartz cuvettes. After equilibration at 37 °C, reactions were initiated by addition of 150 μm NADPH. For reactions of CYP3A4 in the absence of CPR, cumene hydroperoxide was substituted as the electron donor. Product formation rates were determined from the initial linear phase of fluorescence increase. A range of concentrations of 7‐HQ in the same buffer were used to create a standard curve for product concentration, which gave the conversion factor of 16.75 relative fluorescence units·μm^−^ ^1^. Initial velocities were fitted to the Hill equation. NADPH consumption rates, calculated from the absorbance decrease at 340 nm, were measured under the same conditions, and initial velocities were fitted to the Michaelis–Menten equation.

#### Fluorescence detection of superoxide formation using Mito‐HE

Mito‐HE (MitoSOX Red; Thermo Fisher Scientific) was dissolved at 5 mm in DMSO and diluted to 10 μm in 1 mL of 7‐BQ O‐debenzylation reaction mixture (described above), but also containing 1 mg·mL^−1^ salmon sperm DNA to enhance fluorescence of the product, Mito‐2‐hydroxyethidium (2‐OH‐Mito‐E^+^), formed upon reaction of superoxide with Mito‐HE. 7‐BQ O‐debenzylation reactions were then initiated by the addition of NADPH. Production of 2‐OH‐Mito‐E^+^ was monitored by measuring fluorescence emission at 600 nm upon excitation at 527 49, 70.

## Author contributions

KCL and JMXH planned and performed experiments; JMXH wrote the paper; all authors discussed the results and participated in manuscript preparation; SH and NSS initiated and directed this research.

## References

[1] Anzenbacher PI & Anzenbacherová E (2001) Cytochromes P450 and metabolism of xenobiotics. Cell Mol Life Sci 58, 737–747. 11437235 10.1007/PL00000897 PMC11337355

[2] Zanger UM & Schwab M (2013) Cytochrome P450 enzymes in drug metabolism: regulation of gene expression, enzyme activities, and impact of genetic variation. Pharmacol Ther 138, 103–141. 23333322 10.1016/j.pharmthera.2012.12.007

[3] Rendic S & Guengerich FP (2015) Survey of human oxidoreductases and cytochrome P450 enzymes involved in the metabolism of xenobiotic and natural chemicals. Chem Res Toxicol 28, 38–42. 25485457 10.1021/tx500444e PMC4303333

[4] Meunier B , de Visser SP & Shaik S (2004) Mechanism of oxidation reactions catalyzed by cytochrome P450 enzymes. Chem Rev 104, 3947–3980. 15352783 10.1021/cr020443g

[5] Guengerich FP (2007) Mechanisms of cytochrome P450 substrate oxidation: minireview. J Biochem Mol Toxicol 21, 163–168. 17936929 10.1002/jbt.20174

[6] Sligar SG (2010) Glimpsing the critical intermediate in cytochrome P450 oxidations. Science 330, 924–925. 21071657 10.1126/science.1197881 PMC4103181

[7] Cederbaum AI (2015) Molecular mechanisms of the microsomal mixed function oxidases and biological and pathological implications. Redox Biol 4, 60–73. 25498968 10.1016/j.redox.2014.11.008 PMC4309856

[8] Davydov DR (2011) Microsomal monooxygenase as a multienzyme system: the role of P450‐P450 interactions. Expert Opin Drug Metab Toxicol 7, 543–558. 21395496 10.1517/17425255.2011.562194 PMC3079778

[9] Reed JR & Backes WL (2012) Formation of P450‐P450 complexes and their effect on P450 function. Pharmacol Ther 133, 299–310. 22155419 10.1016/j.pharmthera.2011.11.009 PMC3272114

[10] Davydov DR , Davydova NY , Sineva EV & Halpert JR (2015) Interactions among cytochromes P450 in microsomal membranes: oligomerization of cytochromes P450 3A4, 3A5, and 2E1 and its functional consequensces. J Biol Chem 290, 3850–3864. 25533469 10.1074/jbc.M114.615443 PMC4319048

[11] Black SD , French JS , Williams CH & Coon MJ (1979) Role of a hydrophobic polypeptide in the N‐terminal region of NADPH‐cytochrome P‐450 reductase in complex formation with P‐450LM. Biochem Biophys Res Comm 91, 1528–1535. 118758 10.1016/0006-291x(79)91238-5

[12] Black SD & Coon MJ (1982) Structural features of liver microsomal NADPH‐cytochrome P‐450 reductase. Hydrophobic domain, hydrophilic domain, and connecting region. J Biol Chem 257, 5929–5938. 6802823

[13] Black SD (1992) Membrane topology of the mammalian P450 cytochromes. FASEB J 6, 680–685. 1537456 10.1096/fasebj.6.2.1537456

[14] Masters BSS & Okita RT (1980) The history, properties, and function of NADPH‐cytochrome P‐450 reductase. Pharmacol Ther 9, 227–244. 6773077 10.1016/s0163-7258(80)80020-9

[15] Lu AYH & Coon MJ (1968) Role of hemoprotein P‐450 in fatty acid ω‐hydroxylation in a soluble enzyme system from liver microsomes. J Biol Chem 243, 1331–1332. 4385007

[16] Strobel HW , Lu AYH , Heidema J & Coon MJ (1970) Phosphatidylcholine requirement in the enzymatic reduction of hemoprotein P‐450 and in fatty acid, hydrocarbon, and drug hydroxylation. J Biol Chem 245, 4851–4854. 4393962

[17] Baylon JL , Lenov IL , Sligar SG & Tajkhorshid E (2013) Characterizing the membrane‐bound state of cytochrome P450 3A4: structure, depth of insertion, and orientation. J Am Chem Soc 135, 8542–8551. 23697766 10.1021/ja4003525 PMC3682445

[18] Berka K , Paloncýová MT , Anzenbacher P & Otyepka M (2013) Behavior of human cytochromes P450 on lipid membranes. J Phys Chem B 117, 11556−11564. 23987570 10.1021/jp4059559

[19] Navrátilová V , Paloncýová M , Berka K & Otyepka M (2016) Effect of lipid charge on membrane immersion of cytochrome P450 3A4. J Phys Chem B 120, 11205–11213. 27723344 10.1021/acs.jpcb.6b10108

[20] Schleinkofer K , Sudarko , Winn PJ , Lüdemann SK & Wade RC (2005) Do mammalian cytochrome P450s show multiple ligand access pathways and ligand channelling? EMBO Rep 6, 584–589. 16028306 10.1038/sj.embor.7400420 PMC1369091

[21] Fishelovitch D , Shaik S , Wolfson HJ & Nussinov R (2009) Theoretical characterization of substrate access/exit channels in the human cytochrome P450 3A4 enzyme: involvement of phenylalanine residues in the gating mechanism. J Phys Chem B 113, 13018–13025. 19728720 10.1021/jp810386z PMC2750738

[22] Gillam EM , Guo Z , Ueng YF , Yamazaki H , Cock I , Reilly PE , Hooper WD & Guengerich FP (1995) Expression of cytochrome‐P450‐3A5 in *Escherichia coli*: effects of 5′ modification, purification, spectral characterization, reconstitution conditions, and catalytic activities. Arch Biochem Biophys 317, 374–384. 7893152 10.1006/abbi.1995.1177

[23] Cho EY , Yun C‐H , Chae H‐Z , Chae H‐J & Ahn T (2008) Anionic phospholipid‐induced regulation of reactive oxygen species production by human cytochrome P450 2E1. FEBS Lett 582, 1771–1776. 18472009 10.1016/j.febslet.2008.04.048

[24] Jang H‐H , Kim D‐H , Ahn T & Yun C‐H (2010) Functional and conformational modulation of human cytochrome P450 1B1 by anionic phospholipids. Arch Biochem Biophys 493, 143–150. 19857456 10.1016/j.abb.2009.10.012

[25] Brignac‐Huber L , Reed JR & Backes WL (2011) Organization of NADPH‐cytochrome P450 reductase and CYP1A2 in the endoplasmic reticulum—microdomain localization affects monooxygenase function. Mol Pharmacol 79, 549–557. 21156755 10.1124/mol.110.068817 PMC3061359

[26] Brignac‐Huber LM , Reed JR , Eyer MK & Backes WL (2013) Relationship between CYP1A2 localization and lipid microdomain formation as a function of lipid composition. Drug Metab Dispos 41, 1896–1905. 23963955 10.1124/dmd.113.053611 PMC3807054

[27] Ingelman‐Sundberg M , Blanck J , Smettan G & Ruckpaul K (1983) Reduction of cytochrome P‐450 LM2 by NADPH in reconstituted phospholipid vesicles is dependent on membrane charge. Eur J Biochem 134, 157–162. 6407834 10.1111/j.1432-1033.1983.tb07546.x

[28] Imaoka S , Imai Y , Shimada T & Funae Y (1992) Role of phospholipids in reconstituted cytochrome P450‐3A form and mechanism of their activation of catalytic activity. Biochemistry 31, 6063–6069. 1627548 10.1021/bi00141a015

[29] Ahn T , Guengerich FP & Yun C‐H (1998) Membrane insertion of cytochrome P450 1A2 promoted by anionic phospholipids. Biochemistry 37, 12860–12866. 9737864 10.1021/bi980804f

[30] Kim K‐H , Ahn T & Yun C‐H (2003) Membrane properties induced by anionic phospholipids and phosphatidylethanolamine are critical for the membrane binding and catalytic activity of human cytochrome P450 3A4. Biochemistry 42, 15377–15387. 14690448 10.1021/bi035280k

[31] Kim K‐H , Kim D‐H , Jang H‐H , Kim M , Kim D‐H , Kim J‐S , Kim J‐I , Chae HZ , Ahn T & Yun C‐H (2007) Lateral segregation of anionic phospholipids in model membranes induced by cytochrome P450 2B1: bi‐directional coupling between CYP2B1 and anionic phospholipid. Arch Biochem Biophys 468, 226–233. 17980858 10.1016/j.abb.2007.10.003

[32] Ingelman‐Sundberg M , Hagbjörk A‐L , Ueng Y‐F , Yamazaki H & Guengerich FP (1996) High rates of substrate hydroxylation by human cytochrome P450 3A4 in reconstituted membranous vesicles: influence of membrane charge. Biochem Biophys Res Comm 221, 318–322. 8619853 10.1006/bbrc.1996.0593

[33] Eberhart DC & Parkinson A (1991) Cytochrome P450 IIIA1 (P450p) requires cytochrome b5 and phospholipid with unsaturated fatty acids. Arch Biochem Biophys 291, 231–240. 1659320 10.1016/0003-9861(91)90128-6

[34] Nath A , Atkins WM & Sligar SG (2007) Applications of phospholipid bilayer nanodiscs in the study of membranes and membrane proteins. Biochemistry 46, 2059–2069. 17263563 10.1021/bi602371n

[35] Bayburt TH & Sligar SG (2010) Membrane protein assembly into nanodiscs. FEBS Lett 584, 1721–1727. 19836392 10.1016/j.febslet.2009.10.024 PMC4758813

[36] Schuler MA , Denisov IG & Sligar SG (2013) Nanodiscs as a new tool to examine lipid‐protein interactions. Methods Mol Biol 974, 415–433. 23404286 10.1007/978-1-62703-275-9_18 PMC4201044

[37] Das A & Sligar SG (2009) Modulation of the cytochrome P450 reductase redox potential by the phospholipid bilayer. Biochemistry 48, 12104–12112. 19908820 10.1021/bi9011435 PMC2797566

[38] Baas BJ , Denisov IG & Sligar SG (2004) Homotropic cooperativity of monomeric cytochrome P450 3A4 in a nanoscale native bilayer environment. Arch Biochem Biophys 430, 218–228. 15369821 10.1016/j.abb.2004.07.003

[39] Denisov IG , Grinkova YV , Baas BJ & Sligar SG (2006) The ferrous‐dioxygen intermediate in human cytochrome P450 3A4: substrate dependence of formation and decay kinetics. J Biol Chem 281, 23313–23318. 16762915 10.1074/jbc.M605511200

[40] Das A , Grinkova YV & Sligar SG (2007) Redox potential control by drug binding to cytochrome P450 3A4. J Am Chem Soc 129, 13778–13779. 17948999 10.1021/ja074864x PMC2535932

[41] Nath A , Grinkova YV , Sligar SG & Atkins WM (2007) Ligand binding to cytochrome P450 3A4 in phospholipid bilayer nanodiscs: the effect of model membranes. J Biol Chem 282, 28309–28320. 17573349 10.1074/jbc.M703568200

[42] Denisov IG , Baas BJ , Grinkova YV & Sligar SG (2007) Cooperativity in cytochrome P450 3A4: linkages in substrate binding, spin‐state, uncoupling, and product formation. J Biol Chem 282, 7066–7076. 17213193 10.1074/jbc.M609589200

[43] Grinkova YV , Denisov IG , McLean MA & Sligar SG (2013) Oxidase uncoupling in heme monooxygenases: human cytochrome P450 CYP3A4 in nanodiscs. Biochem Biophys Res Comm 430, 1223–1227. 23266608 10.1016/j.bbrc.2012.12.072 PMC4191626

[44] Stresser DM , Turner SD , Blanchard AP , Miller VP & Crespi CL (2002) Cytochrome P450 fluorometric substrates: identification of isoform‐selective probes for rat CYP2D2 and human CYP3A4. Drug Metab Dispos 30, 845–852. 12065444 10.1124/dmd.30.7.845

[45] Ritchie TK , Grinkova YV , Bayburt TH , Denisov IG , Zolnerciks JK , Atkins WM & Sligar SG (2009) Reconstitution of membrane proteins in phospholipid bilayer nanodiscs. Methods Enzymol 464, 211–231. 19903557 10.1016/S0076-6879(09)64011-8 PMC4196316

[46] Mosmann T (1983) Rapid colorimetric assay for cellular growth and survival: application to proliferation and cytotoxicity assays. J Immunol Methods 65, 55–63. 6606682 10.1016/0022-1759(83)90303-4

[47] Guengerich FP , Martin MV , Sohl CD & Cheng Q (2009) Measurement of cytochrome P450 and NADPH‐cytochrome P450 reductase. Nat Protoc 4, 1245–1251. 19661994 10.1038/nprot.2009.121 PMC3843963

[48] Mishin V , Gray JP , Heck DE , Laskin DL & Laskin JD (2010) Application of the Amplex Red/horseradish peroxidase assay to measure hydrogen peroxide generation by recombinant microsomal enzymes. Free Radic Biol Med 48, 1485–1491. 20188819 10.1016/j.freeradbiomed.2010.02.030 PMC3643635

[49] Robinson KM , Janes MS , Pehar M , Monette JS , Ross MF , Hagen TM , Murphy MP & Beckman JS (2006) Selective fluorescent imaging of superoxide *in vivo* using ethidium‐based probes. Proc Natl Acad Sci USA 103, 15038–15043. 17015830 10.1073/pnas.0601945103 PMC1586181

[50] Robinson KM , Janes MS & Beckman JS (2008) The selective detection of mitochondrial superoxide by live cell imaging. Nat Protoc 3, 941–947. 18536642 10.1038/nprot.2008.56

[51] Vermilion JL & Coon MJ (1978) Purified liver microsomal NADPH‐cytochrome P‐450 reductase. Spectral characterization of oxidation‐reduction states. J Biol Chem 253, 2694–2704. 632295

[52] Gum JR & Strobel HW (1979) Purified NADPH cytochrome P‐450 reductase. Interaction with hepatic microsomes and phospholipid vesicles. J Biol Chem 254, 4177–4185. 108270

[53] Venkateswarlu K , Lamb DC , Kelly DE , Manning NJ & Kelly SL (1998) The N‐terminal membrane domain of yeast NADPH‐cytochrome P450 (CYP) oxidoreductase is not required for catalytic activity in sterol biosynthesis or in reconstitution of CYP activity. J Biol Chem 273, 4492–4496. 9468503 10.1074/jbc.273.8.4492

[54] Pudney CR , Khara B , Johannissen LO & Scrutton NS (2011) Coupled motions direct electrons along human microsomal P450 chains. PLoS Biol 9, e1001222. 22205878 10.1371/journal.pbio.1001222 PMC3243717

[55] Hedison TM , Hay S & Scrutton NS (2015) Real‐time analysis of conformational control in electron transfer reactions of human cytochrome P450 reductase with cytochrome c. FEBS J 282, 4357–4375. 26307151 10.1111/febs.13501 PMC4973710

[56] Glaumann H & Dallner G (1968) Lipid composition and turnover of rough and smooth microsomal membranes in rat liver. J Lipid Res 9, 720–729. 5685264

[57] Colbeau A , Nachbaur J & Vignais PM (1971) Enzymic characterization and lipid composition of rat liver subcellular membranes. Biochim Biophys Acta 249, 462–492. 5134192 10.1016/0005-2736(71)90123-4

[58] Lee AG (2004) How lipids affect the activities of integral membrane proteins. Biochim Biophys Acta 3, 1–2. 10.1016/j.bbamem.2004.05.012 15519309

[59] Lange C , Nett JH , Trumpower BL & Hunte C (2001) Specific roles of protein–phospholipid interactions in the yeast cytochrome bc1 complex structure. EMBO J 20, 6591–6600. 11726495 10.1093/emboj/20.23.6591 PMC125751

[60] Grover TA & Piette LH (1981) Influence of flavin addition and removal on the formation of superoxide by NADPH‐cytochrome P‐450 reductase: a spin‐trap study. Arch Biochem Biophys 212, 105–114. 6272650 10.1016/0003-9861(81)90348-9

[61] Marohnic CC , Panda SP , Martasek P & Masters BS (2006) Diminished FAD binding in the Y459H and V492E Antley‐Bixler syndrome mutants of human cytochrome P450 reductase. J Biol Chem 281, 35975–35982. 16998238 10.1074/jbc.M607095200

[62] Shen AL , Porter TD , Wilson TE & Kasper CB (1989) Structural analysis of the FMN‐binding domain of NADPH‐cytochrome P‐450 oxidoreductase by site‐directed mutagenesis. J Biol Chem 264, 7584–7589. 2708380

[63] Ghrayeb J , Kimura H , Takahara M , Hsiung H , Masui Y & Inouye M (1984) Secretion cloning vectors in *Escherichia coli* . EMBO J 3, 2437–2442. 6094184 10.1002/j.1460-2075.1984.tb02151.x PMC557705

[64] Oprian DD & Coon MJ (1982) Oxidation‐reduction states of FMN and FAD in NADPH‐cytochrome P‐450 reductase during reduction by NADPH. J Biol Chem 257, 8935–8944. 6807985

[65] Smith GC , Tew DG & Wolf CR (1994) Dissection of NADPH‐cytochrome P450 oxidoreductase into distinct functional domains. Proc Natl Acad Sci USA 91, 8710–8714. 8078947 10.1073/pnas.91.18.8710 PMC44676

[66] Pritchard M , McLaughlin L & Friedberg T (2006) Establishment of functional human cytochrome P450 monooxygenase systems in *Escherichia coli* In Cytochrome P450 Protocols (Phillips IR & Shephard EA, eds), pp. 19–29. Humana Press Inc., Totowa, NJ. 10.1385/1-59259-998-2:19 16719371

[67] Barr I & Guo F (2015) Pyridine hemochromagen assay for determining the concentration of Heme in purified protein solutions. Bio Protoc 5, e1594. 10.21769/bioprotoc.1594 PMC4932910 27390766

[68] Berry EA & Trumpower BL (1987) Simultaneous determination of hemes a, b, and c from pyridine hemochrome spectra. Anal Biochem 161, 1–15. 3578775 10.1016/0003-2697(87)90643-9

[69] Bligh EG & Dyer WJ (1959) A rapid method of total lipid extraction and purification. Can J Biochem Physiol 37, 911–917. 13671378 10.1139/o59-099

[70] Zielonka J & Kalyanaraman B (2010) Hydroethidine‐ and Mito‐SOX‐derived red fluorescence is not a reliable indicator of intracellular superoxide formation: another inconvenient truth. Free Radic Biol Med 48, 983–1001. 20116425 10.1016/j.freeradbiomed.2010.01.028 PMC3587154

